# Necrotizing Soft Tissue Infection and Perforated Viscus After Suction-Assisted Lipectomy

**DOI:** 10.7759/cureus.8617

**Published:** 2020-06-14

**Authors:** Justin Rafael O De la Fuente, Anwar Ferdinand, Matthew Dybas, Tim Montrief, Jorge Cabrera

**Affiliations:** 1 Critical Care Medicine, University of Miami Miller School of Medicine, Miami, USA; 2 Emergency Medicine, Jackson Memorial Hospital, Miami, USA; 3 Critical Care Medicine, University of Pittsburgh Medical Center, Pittsburgh, USA; 4 Pulmonary and Critical Care Medicine, University of Miami Miller School of Medicine, Miami, USA

**Keywords:** necrotizing soft tissue infections, nsti, necrotizing fasciitis, antibiotics, liposuction, cosmetic surgery, complication, plastic surgery, perforated viscus

## Abstract

Suction-assisted lipectomy (SAL) is a commonly performed cosmetic surgery in the United States and has been steadily increasing in popularity over the past few years. As more of these surgeries are performed, several rare but life-threatening complications are being recognized, including necrotizing soft tissue infections (NSTIs). NSTIs require rapid surgical intervention but can be challenging to diagnose, as skin manifestations may be difficult to differentiate from normal post-SAL changes. We present a case of a 44-year-old female who presented with signs of septic shock after SAL of her abdomen and back. She was ultimately found to have an NSTI of her abdominal wall, likely due to perforated viscus that occurred as a complication of her procedure. This case demonstrates the significance of recognizing NSTIs as a potential complication of SAL in ill-appearing patients with non-specific symptoms and septic shock.

## Introduction

Suction-assisted lipectomy (SAL), also known as liposuction, is one of the most popular cosmetic procedures in the United States, with more than 250,000 performed in 2018 [1]. While SAL is widely considered a minor surgery, it is not without potentially serious risks including perforated viscus, local anesthetic systemic toxicity, intravascular fluid shifts, hematoma formation, skin necrosis, and surgical site infections [2,3]. A rare but important complication that has been associated with liposuction is necrotizing fasciitis, which is a dangerous and rapidly progressive infection of the soft tissue [3,4]. This condition has many causes ranging from minor skin lacerations to major surgical procedures, and clinical manifestations typically include soft-tissue swelling, skin bullae, and erythema [5,6]. These cutaneous findings are often absent until late in the disease progression, and other less specific manifestations such as pain out of proportion on the physical examination, fever, and fatigue can complicate the diagnosis of necrotizing fasciitis, particularly in the context of a recent surgery where symptoms may mimic routine post-surgical changes [6,7]. The majority of necrotizing infections identified due to liposuction are monomicrobial and associated with *Streptococcus pyogenes* [3]. Here, we describe a rare case of iatrogenic perforated viscus with *Escherichia coli* necrotizing fasciitis in a patient after SAL.

## Case presentation

A 44-year-old female with a medical history of hypertension and gastric sleeve surgery presented to the emergency department complaining of generalized abdominal pain. She had recently undergone a SAL of her abdomen and bilateral flanks eight days prior to evaluation. Associated symptoms included fever, nausea, and multiple episodes of vomiting. Review of systems was otherwise unremarkable.

Initial vital signs were notable for a temperature of 100.4°F, a blood pressure of 92/50 mm/Hg, a respiratory rate of 24 breaths per minute, a heart rate of 126 beats per minute, and an oxygen saturation of 99% on room air. On physical examination, there was diffuse abdominal tenderness with rebound and voluntary guarding, but the overlying skin was unremarkable for bullae or skin necrosis. However, there was mild crepitus throughout the abdominal wall. She had multiple surgical incisions across her bilateral flanks and abdominal wall draining minimal serosanguinous fluid.

**Figure 1:**
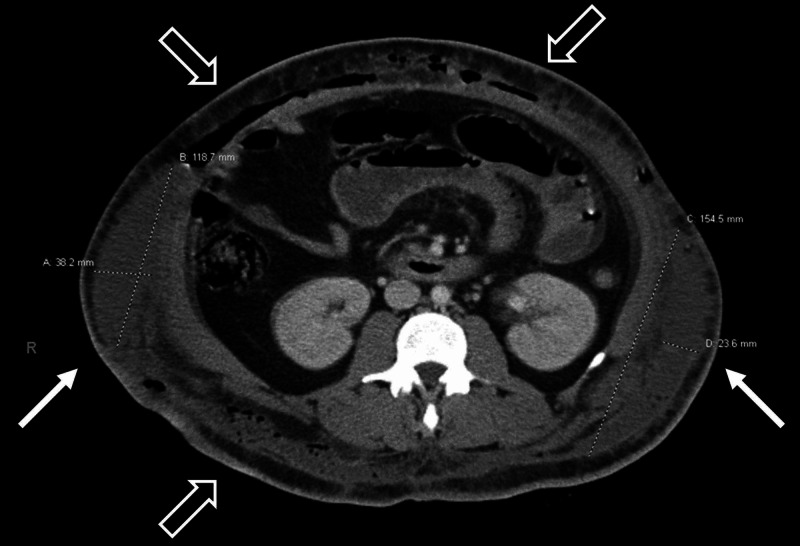
Transverse CT image showing bilateral flank fluid collections (white arrows) measuring approximately 21 x 3.8 x 12cm on the right and 15 x 2.4 x 15.5cm on the left. Numerous foci of subcutaneous emphysema can also be seen (blank arrows).

**Figure 2:**
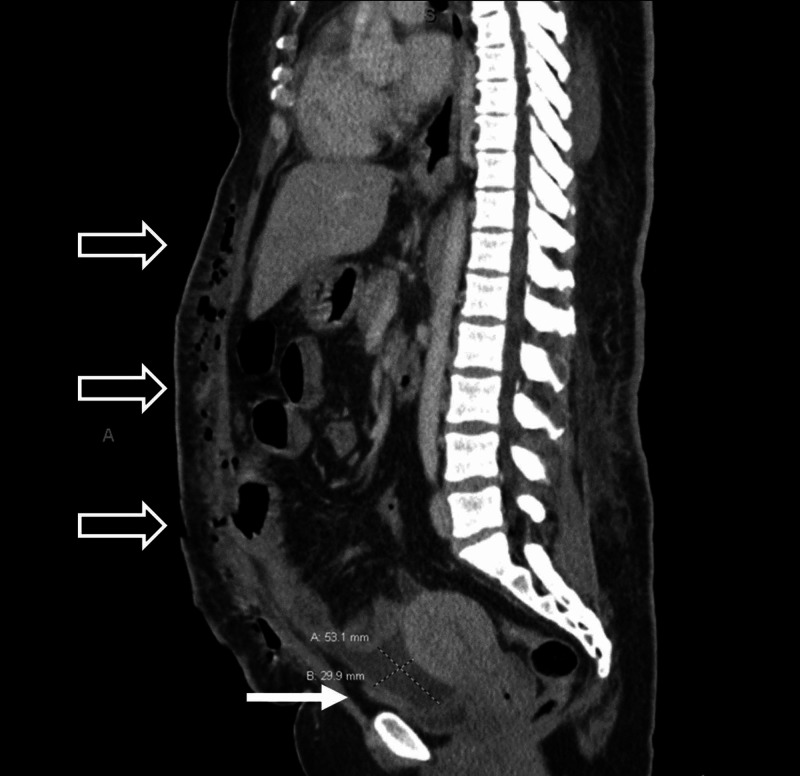
Sagittal CT image showing an intrapelvic fluid collection anterior to the uterus measuring approximately 5.3 x 3.0 x 6.9 cm (white arrow). Numerous foci of subcutaneous emphysema can also be seen (blank arrows).

**Figure 3:**
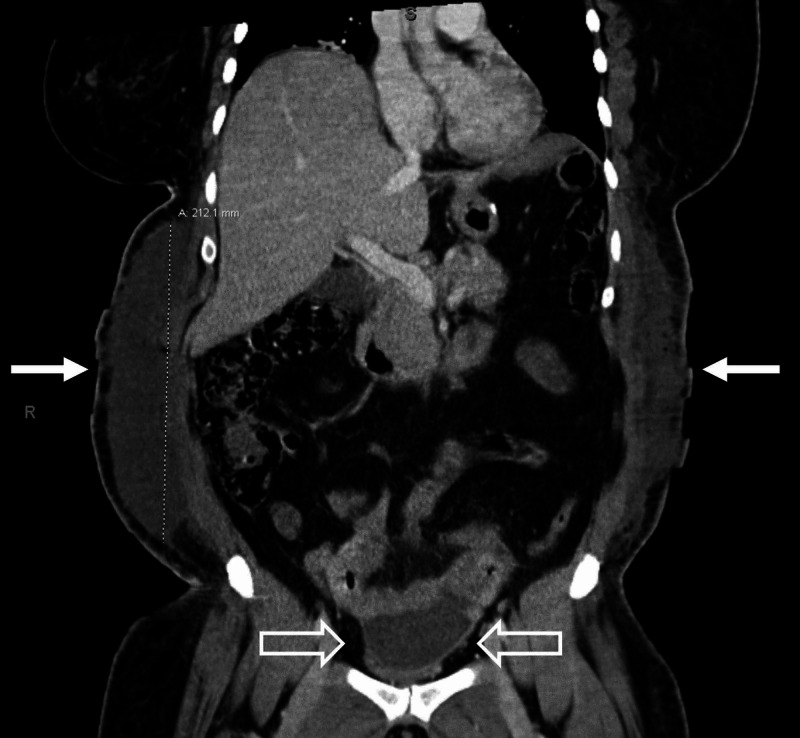
Coronal CT image showing bilateral flank fluid collections (white arrows) and an intraperitoneal fluid collection (blank arrows).

Labs were significant for a white blood cell count of 17.1 x 10^3^/mcL, hemoglobin of 7.9 g/dL, creatinine of 1.57 mg/dL, and lactic acid of 2.9 mmol/L. Arterial blood gas revealed a primary respiratory alkalosis with secondary metabolic acidosis. The patient's pH was 7.52, with PCO_2_ of 22 mmHg, PO_2_ of 162 mmHg, and HCO_3_ of 17. Due to her physical examination findings and recent abdominal surgery, a CT scan with intravenous contrast of the abdomen and pelvis was ordered, which demonstrated fluid collections measuring approximately 21 x 3.8 x 12 cm on the right flank and 15 x 2.4 x 15.5 cm on the left flank (Figure 1), as well as an intrapelvic fluid collection anterior to the uterus measuring 5.3 x 3.0 x 6.9 cm (Figure 2). Other findings included diffuse subcutaneous emphysema and soft tissue stranding of the abdominal wall overlying the fascial planes (Figures 2, 3).

Based on the initial physical examination, hemodynamic instability, and recent abdominal surgery, broad-spectrum antibiotics were administered intravenously due to concern for necrotizing soft tissue infection (NSTI) before imaging was obtained. At this time, the patient received 2 liters of lactated ringers, with improvement in blood pressure and heart rate. Acute care surgery team was consulted and the patient was taken for exploratory laparotomy, which confirmed the diagnosis of necrotizing fasciitis as well as several mesenteric abscesses and seven small bowel enterotomies. Extensive debridement was performed with abscess drainage, enterotomy repairs, and an abdominal washout, followed by temporary closure and close monitoring in the surgical intensive care unit. Final abdominal wall and flank abscess wound cultures grew pan-sensitive E. coli. She underwent several follow-up surgeries for further debridement and abdominal washout followed by wound vacuum placement during her complicated 31-day hospitalization.

## Discussion

SAL is one of the most commonly performed cosmetic surgeries, and providers must be aware of surgical technique as well as potential complications [3]. Outpatient SAL is typically performed under local anesthesia and is used commonly on the buttocks, back, thighs, face, chest, and abdomen. The predominant technique, *microcannula tumescent liposuction*, consists of suction removal of fat from deep subcutaneous layers through aspiration cannulae introduced through small skin incisions [8]. Several liters of tumescent solution consisting of dilute local anesthetic, epinephrine, and crystalloid are infiltrated into the subcutaneous tissue, percolating through tissue layers prior to aspiration [9]. The saline makes the subcutaneous tissue firm and turgid from absorption (tumescence), epinephrine causes vasoconstriction which decreases bleeding, and lidocaine induces local anesthesia [10]. Generally, incisions are left open to drain any remaining fluid [11].

Major, potentially life-threatening complications such as infection, pulmonary embolism, and skin necrosis complicate between 0.02 and 0.25% of all SAL procedures [12]. NSTIs are exceedingly rare in this population, with a 2017 systematic review on the topic revealing that of 3,782 papers discussing necrotizing fasciitis, only 10 involved NSTI associated with SAL [3]. Likewise, Cárdenas-Camarena reported a 0.09% incidence of surgical site infection, with only one case of infection in 1,047 patients who underwent SAL [13,14].

Necrotizing fasciitis is a life-threatening soft tissue infection characterized by a rapid progression of disease. Numerous studies have established the importance of early surgical intervention to reduce morbidity and mortality [6]. As such, a high index of suspicion is needed for timely identification and treatment of these infections. Delays in diagnosis are common however, with one study involving 89 individuals reporting that only around 15% of patients received an accurate diagnosis of NSTI by the time of hospital admission [15]. Being aware of clinical scenarios such as SAL, in which this disease may arise, as well as its manifestations, can help ensure providers accurately diagnose this emergent condition.

The clinical manifestations of NSTI may vary widely, but in patients who present with signs of skin infection in the context of a recent surgery such as SAL, necrotizing infection must be considered [6]. Necrotizing fasciitis often lacks obvious cutaneous manifestations early in the disease course, as was the case in this 44-year-old female. Early, non-specific manifestations of NSTI including incisional pain, fever, and fatigue can easily be misattributed to post-surgical changes [6,16]. Physical examination may reveal severe pain out of proportion, edema extending beyond erythema, and skin bullae [6]. However, many patients present with similar symptoms due to residual tumescent anesthesia, lymphedema, hematoma, or post-operative collections [9,17].

Diagnosis of necrotizing fasciitis is based on history and clinical examination but often requires surgery to confirm. Imaging is another modality that is very useful in the evaluation of patients with suspected necrotizing fasciitis. CT with IV contrast in particular has been found to be a rapid and highly sensitive modality that helps with diagnosis and evaluation of the extent of the disease [18]. Some potential findings include thickening or non-enhancement of the fascia, fluid or gas collections dissecting a fascial plane, and gas involving soft tissue or muscle [3,18]. Ultrasound may also be a useful bedside tool, particularly to distinguish necrotizing infection from cellulitis, as the latter is less likely to reveal subcutaneous thickening or fluid accumulation [19]. Importantly, if clinical suspicion for a necrotizing infection is high, imaging should not delay surgical intervention [6].

In addition to emergent surgical intervention for proper infection source control, initial management should focus on aggressive resuscitation and empiric antibiotics [5,6]. Patients require intensive care for metabolic support, analgesia, and prompt fluid resuscitation with vasopressors as indicated [6]. For empiric antibiotics, current guidelines initially recommend broad-spectrum coverage including methicillin-resistant *Staphylococcus aureus *and gram-negative organisms [5,20]. Although the majority of cases of necrotizing fasciitis due to SAL are monomicrobial with *S. pyogenes*, this case of a 44-year-old female illustrates the importance of broad coverage. Her final wound cultures came back positive for *E. coli*, which were likely related to the small bowel perforation. The recommended therapy for S. pyogenes necrotizing fasciitis, clindamycin with penicillin, would have had limited effectiveness [6].

## Conclusions

As the popularity of SAL continues to grow, providers must maintain a high clinical suspicion in order to differentiate normal post-surgical changes or uncomplicated cellulitis from life-threatening necrotizing fasciitis. Management for this condition is time-sensitive, and early diagnosis is key to ensuring appropriate interventions are made as soon as possible.

## References

[1] American Society of Plastic Surgeons (2020). 2018 Plastic Surgery Statistics Report: American Society of Plastic Surgeons. Plastic Surgery Statistics Report.

[2] Cárdenas-Camarena L, Andrés Gerardo L-P, Durán H, Bayter-Marin JE (2017). Strategies for reducing fatal complications in liposuction. Plast Reconstr Surg Glob Open.

[3] Marchesi A, Marcelli S, Parodi PC, Perrotta RE, Riccio M, Vaienti L (2017). Necrotizing fasciitis in aesthetic surgery: a review of the literature. Aesthetic Plast Surg.

[4] Dellière V, Bertheuil N, Harnois Y, Thiénot S, Gérard M, Robert M, Watier E (2014). Multiple bowel perforation and necrotising fasciitis secondary to abdominal liposuction in a patient with bilateral lumbar hernia. Indian J Plast Surg.

[5] Bonne SL, Kadri SS (2017). Evaluation and management of necrotizing soft tissue infections. Infect Dis Clin North Am.

[6] Stevens DL, Bryant AE (2017). Necrotizing soft-tissue infections. N Engl J Med.

[7] Sarchi A, Long B (2020). A nearly missed case of severe necrotizing soft tissue infection. Cureus.

[8] Venkataram J (2008). Tumescent liposuction: a review. J Cutan Aesthet Surg.

[9] Matarasso A, Levine SM (2013). Evidence-based medicine: liposuction. Plast Reconstr Surg.

[10] Manassa EH, Hellmich S, Ronert M, Hofheinz H, Olbrisch RR Pain management after lipoplasty: a study of 303 cases. Plast Reconstr Surg.

[11] Bellini E, Grieco MP, Raposio E (2017). A journey through liposuction and liposculture: review. Ann Med Surg (Lond).

[12] You JS, Chung YE, Baek SE, Chung SP, Kim MJ (2015). Imaging findings of liposuction with an emphasis on postsurgical complications. Korean J Radiol.

[13] Cárdenas-Camarena L (2003). Lipoaspiration and its complications: a safe operation. Plast Reconstr Surg.

[14] Kaoutzanis C, Gupta V, Winocour J, Shack B, Grotting JC, Higdon K (2017). Incidence and risk factors for major surgical site infections in aesthetic surgery: analysis of 129,007 patients. Aesthet Surg J.

[15] Goh T, Goh LG, Ang CH, Wong CH (2014). Early diagnosis of necrotizing fasciitis. Br J Surg.

[16] Barillo DJ, Cancio LC, Kim SH, Shirani KZ, Goodwin CW (1998). Fatal and near-fatal complications of liposuction. South Med J.

[17] Ezzeddine H, Husari A, Nassar H, Kanso M, El Nounou G, Khalife M, Faraj W (2018). Life threatening complications post-liposuction. Aesthetic Plast Surg.

[18] Chaudhry AA, Baker KS, Gould ES, Gupta R (2015). Necrotizing fasciitis and its mimics: what radiologists need to know. AJR Am J Roentgenol.

[19] Lin CN, Hsiao CT, Chang CP, Huang TY, Hsiao KY, Chen YC, Fann WC (2019). The relationship between fluid accumulation in ultrasonography and the diagnosis and prognosis of patients with necrotizing fasciitis. Ultrasound Med Biol.

[20] Stevens DL, Bisno AL, Chambers HF (2014). Practice guidelines for the diagnosis and management of skin and soft tissue infections: 2014 update by the Infectious Diseases Society of America.

